# Response time models separate single- and dual-process accounts of memory-based decisions

**DOI:** 10.3758/s13423-020-01794-9

**Published:** 2020-09-28

**Authors:** Peter M. Kraemer, Laura Fontanesi, Mikhail S. Spektor, Sebastian Gluth

**Affiliations:** 1 Department of Psychology, University of Basel, Missionsstrasse 62a, 4055 Basel, Switzerland; 2 Universitat Pompeu Fabra, Ramon Trias Fargas, 25, Barcelona, 08005 Spain; 3 Barcelona Graduate School of Economics, Ramon Trias Fargas, 25, Barcelona, 08005 Spain

**Keywords:** Judgment, Decision making

## Abstract

Human decisions often deviate from economic rationality and are influenced by cognitive biases. One such bias is the *memory bias* according to which people prefer choice options they have a better memory of—even when the options’ utilities are comparatively low. Although this phenomenon is well supported empirically, its cognitive foundation remains elusive. Here we test two conceivable computational accounts of the memory bias against each other. On the one hand, a single-process account explains the memory bias by assuming a single biased evidence-accumulation process in favor of remembered options. On the contrary, a dual-process account posits that some decisions are driven by a purely memory-driven process and others by a utility-maximizing one. We show that both accounts are indistinguishable based on choices alone as they make similar predictions with respect to the memory bias. However, they make qualitatively different predictions about response times. We tested the qualitative and quantitative predictions of both accounts on behavioral data from a memory-based decision-making task. Our results show that a single-process account provides a better account of the data, both qualitatively and quantitatively. In addition to deepening our understanding of memory-based decision-making, our study provides an example of how to rigorously compare single- versus dual-process models using empirical data and hierarchical Bayesian parameter estimation methods.

## Introduction

Many decisions in our daily lives, such as where to go on holiday or what to buy in a grocery store, rely on information from memory. Although the role of memory processes in judgements and decision-making has been neglected for a long time, researchers have recently put more emphasis on the relation of these two domains (Shadlen & Shohamy, 2016; Weilbächer & Gluth, 2017). For example, recent studies focused on how memory, by playing a role in the evaluation process, can contribute to violations of standard economic theories of decision-making (Weber & Johnson, 2006).

**Fig. 1:**
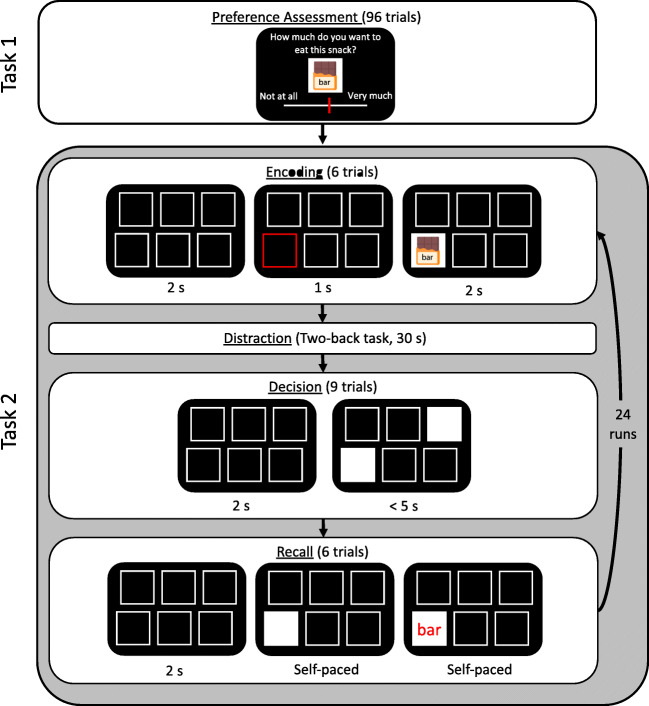
Experiment on memory-based decision-making. During task 1, participants rated their subjective value for all snacks. Task 2 was the remember-and-decide task which comprised four phases: encoding, distraction, decision and recall. Here, we show one example trial per phase. During encoding, participants associated snacks with individual locations. The distraction phase contained a 2-back task with integers. In the decision phase, they retrieved snack-location associations from memory and made preferential choices between snacks. During recall, participants indicated which snack they associated with each location. Note that this is a simplified depiction of the experiment. For a full overview, see Mechera-Ostrovsky and Gluth (2018)

Gluth et al., (2015) showed one of such violations in a decision-making task in which the subjective value (utility) of the options had to be recalled from memory. In this paradigm, individuals first learn to associate different snacks with specific locations. Afterward, they choose between two locations and therefore need to remember to which snacks the two locations were associated with (see Fig. 1). The authors reported that participants tended to prefer remembered snacks over forgotten snacks, even when the subjective value of the former was lower than average (and thus more likely to be lower than the forgotten option’s subjective value). The authors referred to this effect as the *memory bias*. Using functional magnetic resonance imaging (fMRI), Gluth et al., (2015) further showed that this tendency was mediated by an increased effective connectivity from the hippocampus to the ventromedial prefrontal cortex. Since these areas are typically associated with memory and value-based decisions, respectively, the fMRI results supported the idea that memory processes exert a biasing influence on valuation and choice processes. Follow-up studies replicated the memory bias and found that it was partly driven by beliefs about the dependency of memory strength on utility (Mechera-Ostrovsky and Gluth, 2018), and that it exhibited typical characteristics of decisions under uncertainty (Weilbächer et al., in press).

Gluth et al., (2015) proposed a computational model that assumes people to choose between a remembered and a forgotten option by comparing the remembered option’s value against a reference value. If this reference value is below the average snack value, the model predicts that people are more likely to choose remembered options, leading to the memory bias. Critically, this account assumes that all decisions between a remembered and a forgotten option result from the very same comparison-against-reference-value process. Therefore, it is a *single-process account* of the memory bias. Gluth et al., (2015)’s account of the memory bias is thus in stark contrast to dual-process theories (Kahneman and Frederick, 2002; Evans, 2008). Dual-process models assume that decisions are driven by two independent processes (so-called “type 1” and “type 2” processes) (Evans & Stanovich, 2013). Type 1 processes are described as intuitive processes that lead to relatively fast, automatic, and uncontrollable choices. Type 2 processes, on the other hand, are controlled, deliberate processes that lead to slower responses that are closer to normative predictions. Type 2 processes are thus viewed as rational processes (but see Oaksford & Hall, 2016). Such a *dual-process account* explains the memory bias as follows: In some cases, people make a decision based on a type 1 process which leads them to choose the option they remember better–intuitively and independently of its subjective value. In other cases, they make a decision based on a type 2 process. The type 2 process implements an unbiased choice, based on a cognitively demanding decision process that takes the subjective value of the remembered option into account in a rational (i.e., utility-maximizing) way.

As we will show, both single- and dual-process accounts can produce the memory bias on choice. Thus, we face a model-selection problem: Two models can account for the same behavioral phenomenon, but the assumed underlying cognitive processes are fundamentally different. To find out which model is more suitable to explain the memory bias, we consider an additional data dimension, namely response times (RTs). The consideration of RTs has a rich tradition in psychological research, since they contain information about the underlying cognitive processes (Luce, 1986). Additionally, RTs can aid model selection (Ballard & McClure, 2019; Gluth & Meiran, 2019; Wilson & Collins, 2019). Critically, we will show that although the single- and dual-process accounts can make similar predictions on choice behavior, they differ with respect to RTs. Therefore, considering both dimensions, choices and RTs, aids to resolve the present model-selection problem.

**Fig. 2:**
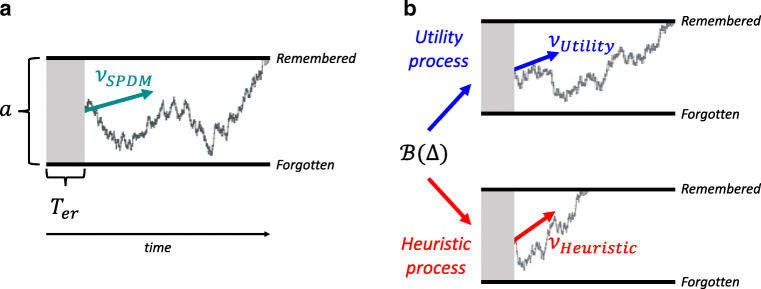
Diffusion decision models. **a** Diffusion process of the single-process account. Evidence for an option accumulates over time with a rate of *ν*_*SPDM*_ until the threshold (boundary) for one choice option (remembered or forgotten) is reached. The boundary separation depends on the parameter *a*, and *T*_*er*_ depicts the non-decision time. **b** The dual-process account assumes two diffusion processes with different drift rates (*ν*_*Utility*_ and *ν*_*Heuristic*_). A Bernoulli trial based on the parameter Δ selects either the utility or the heuristic diffusion process

Joint modeling of choices and RTs is often done in the framework of sequential-sampling (or evidence-accumulation) models (Bogacz et al., 2006). A popular sequential-sampling model is the diffusion decision model (DDM) (Ratcliff, 1978; Ratcliff & Rouder, 1998). In a nutshell, the DDM describes a decision between two options as an accumulation of noisy, relative evidence over time (see also Fig. 2). Evidence, in the current task, represents the information regarding an option’s subjective value that is recalled from memory. The accumulation process ends when the relative evidence for one or the other option surpasses a certain threshold. At that point, the decision is made. The accumulation process can also be biased towards one of the two options already at the beginning of a trial. The rate of evidence accumulation is referred to as the drift rate of the decision process. The higher the drift rate towards a specific option, the more often that option is chosen and the faster the decision. On the other side, the threshold controls how cautiously decisions are made. The higher the threshold, the slower and more consistent the decisions are.

Over the years, the DDM has been applied successfully to processes of memory retrieval and perceptual decision-making (Ratcliff et al., 2016), but researchers also adapted it to value-based decisions (for recent overviews see Busemeyer et al., (2019) and Clithero, 2018). Importantly, Gluth et al. (2015) used the DDM as the basis of their single-process account of memory-based decisions. Within their model,the memory bias results from abiased reference value for forgotten options, which influences the drift rate of the decision process. In recent years, novel evidence-accumulation models were developed to reflect ideas from dual-process theories of decision-making (Diederich & Trueblood, 2018; Alós-Ferrer, 2018). In particular, the dual-process diffusion model by Alós-Ferrer provided a suitable account of a range of phenomena from judgment and decision-making tasks. The model assumes two independent decision processes: a type 1-like heuristic process and a type 2-like utility-maximizing process. Since both the single- and dual-process accounts operate within the same framework (i.e., the DDM), it is possible to rigorously evaluate which of the two accounts better explains the memory bias.

In the present study, we first derived choice and RT predictions of a single- and a dual-process DDM in the context of memory-based decisions. Next, we compared the different qualitative predictions of these models with the actual RT data from a comparatively large sample of participants who performed the remember-and-decide task (Mechera-Ostrovsky & Gluth, 2018). Finally, we conducted a quantitative model comparison within a hierarchical Bayesian parameter estimation framework. Both our qualitative and quantitative comparisons lend consistent support for the single-process account, thus strengthening our knowledge of the computational cognitive basis of value-based decisions from memory.

## Methods

### Participants

We analyzed data from a previously published study (Mechera-Ostrovsky & Gluth, 2018). In total, 96 participants (67 female, mean age = 23.5, age range: [19,35]) took part in that study. Due to early termination and age restrictions, the data from six participants were not analyzed. The participants were students who took part in the study for course credits. The procedure was approved by the ethics committee of the University of Basel and all participants gave written informed consent.

### Procedures

The full experimental procedure is described in Mechera-Ostrovsky and Gluth (2018). Here, we summarize the procedures relevant to the present research question. Participants were required to fast for four hours before the experiment started. They familiarized themselves with a set of 48 snacks. For each snack, they learned intuitive three-letter abbreviations (e.g., “sni” for “Snickers”) until they reached 100% accuracy. Participants’ subjective valuation of each snack was assessed on a continuous rating scale (Fig. 1). This evaluation was incentivized by selecting two snacks randomly at the end of the experiment and giving the higher-rated snack to the participant to eat. After eliciting subjective valuations, participants faced the remember-and-decide task which consisted of the four periods encoding, distraction, decision, and recall (in that order). During encoding, participants saw empty squares at six different locations on the screen. One after another, each location was highlighted, and a snack image appeared in the respective square. Participants had to associate and remember which snack was located in which square. During the distraction period, participants performed a 2-back working memory task that prevented them from rehearsing the information obtained in the encoding phase. During the decision period, the six squares were presented again and, in each trial, two squares were highlighted. Participants picked one of the two snacks hidden behind the empty squares. Since the snack identity was not visually accessible, they had to retrieve it from memory to make an informed choice. During recall, the snack-location associations were probed to assess memory strength for each snack location.

### Data preprocessing

First, we excluded trials that were unlikely to originate from a deliberate process: In particular, we excluded trials in which no choice was made (2.0*%*) and in which RTs were lower than 200 ms (1.4*%*). We then excluded trials that do not help to discriminate between single- and dual-process accounts: In particular, both accounts make the same predictions for behavior in trials in which both options are remembered or both options are forgotten. Therefore, we restricted our analyses to trials where one snack was remembered and the other was forgotten. This resulted in a total of 8031 trials (on average 89.2 trials per participant, *SD* = 15.80, range: [39,118]). See Appendix A1 for more information on the trial types and Appendix B3 for analyses including all trial types.

### Cognitive models

#### The diffusion decision model

In its original form, the DDM predicts choices and RTs using four parameters (Ratcliff, 1978). First, the boundary separation *a* determines the (relative) amount of evidence required to terminate the deliberation process. This parameter is responsible for speed–accuracy tradeoffs. Second, the starting-point bias *z* determines the amount of relative evidence at the beginning of the deliberation process. This parameter reflects prior information or a bias in favor of one of the options. Third, the drift rate *ν* determines the speed of evidence accumulation. In value-based decision-making, the drift rate is often directly proportional to the value difference between the two available options (e.g., Krajbich et al.,, 2010). The stronger the value difference, the higher the drift rate, making choices both faster and more frequently in favor of the option the drift rate is directed to. Analogously, small differences of values imply a low drift rate and thus higher RTs and less frequent choices in the direction of the drift rate. Finally, the non-decision time *T*_*er*_ absorbs every process that is not part of the deliberation process, such as the time it takes to execute the button press or to visually encode the stimuli.

#### Single-process account

According to Gluth et al., (2015)’s model of the memory bias, participants compare the subjective value of the remembered snack with a reference value. Thus, the drift rate depends on a comparison process such that

1 $$  \nu_{SPDM} = V_{rem}-\gamma, $$

where *ν*_*SPDM*_ is the drift rate, *V*_*rem*_ is the subjective value of the remembered option and *γ* is the reference value. Here, a single evaluation process gives rise to the memory bias in every trial. This is why we refer to the model as a single-process diffusion model (SPDM). An example of a diffusion process is depicted in Fig. 2a. Importantly, Gluth et al., (2015) argued that—assuming that memory strength is independent of value—this reference value should be unbiased (i.e., equal to the mean of all options) in order to maximize utility. When estimating it as a free parameter, however, the reference value was found to be biased such that remembered options appeared to be more valuable, even if they were comparatively unattractive.

#### Dual-process account

To model the dual-process account, we adopted the dual-process diffusion model (DPDM) by Alós-Ferrer (2018). The DPDM assumes that people vary between two types of choice strategies across trials (see Fig. 2b). In some trials, people use a *utility process* which captures “computational-normative aspects of decision-making” (p. 203). In other trials, a *heuristic process* favors “intuitive-affective attributes” (ibid) of a choice option with a relatively high drift rate, leading to fast and more consistent responses. The selection of a process in a given trial is supposedly governed by the central executive. It selects the utility process with the probability Δ and the heuristic process with 1 −Δ. The drift rate of the DPDM in a given trial is given by

2 $$ \nu_{DPDM}= \begin{cases} \nu_{Utility}, & \text{if}\ k=1 \\ \nu_{Heuristic}, & \text{if}\ k=0\\ \end{cases} $$

where *k* is the outcome of a Bernoulli trial ${\mathscr{B}}({\Delta })$.

In the context of memory-based decisions in the remember-and-decide task, we propose that *ν*_*Utility*_ reflects the utility-maximizing process in which *V*_*rem*_ is compared to the (unbiased) average snack value of all possible snacks *V*_*avg*_. Thus, the cognitively demanding utility process tends to select the option with the higher subjective utility without a bias towards the remembered option. If individuals do not follow the utility-maximizing process, they rely on a simple decision rule to make a choice. In the context of memory-based decisions, they can use recognition as a cue for value, much in line with the recognition heuristic in judgment tasks (Goldstein and Gigerenzer, 2002) (see also Discussion). In the diffusion-model framework, *ν*_*Heuristic*_ reflects the drift rate of the heuristic process that favors the choice of a snack, because it can be recalled correctly. Importantly, *ν*_*Heuristic*_ is independent of the snack’s subjective value.

### Qualitative predictions

The SPDM and DPDM can make very similar predictions regarding choices, but they differ significantly with respect to their predictions of RTs.

Previous research (e.g., Bogacz et al.,, 2006) showed that, in a simplified version of the DDM (i.e., without the starting-point bias and without across-trial variability in any of the parameters), choices of option *A* over *B* are related to the DDM parameters as

3 $$ P(\text{choose A} | \nu, a, \sigma) = 1 - {\frac {1}{1+e^{\frac{2\nu a}{\sigma^{2}}}}}, $$

where *ν* is the drift rate, *a* is the boundary separation, and *σ* is the noise of the drift process. If $\nu \to \infty $, participants are more likely to select option *A* and, conversely, if $\nu \to -\infty $, participants are more likely to choose option *B*. If *ν* = 0, participants are indifferent between *A* and *B*.

The expected RTs under the DDM are given by

4 $$ \begin{array}{@{}rcl@{}} && RT = T_{er} + DT, \\ && DT(\nu, a, \sigma) = {\frac{a}{\nu}\tanh{(\frac {\nu a}{\sigma^{2}})}}, \end{array} $$

where *T*_*er*_ is the non-decision time and DT is the mean decision time as a function of the diffusion parameters (Bogacz et al., 2006). The DDM typically predicts an inverted U-shaped curve of the average RTs as a function of the drift: The expected RTs are slow when the speed of evidence accumulation is low (*ν* = 0) and fast when the speed of evidence accumulation is high ($\nu \to \pm \infty $).

**Fig. 3:**
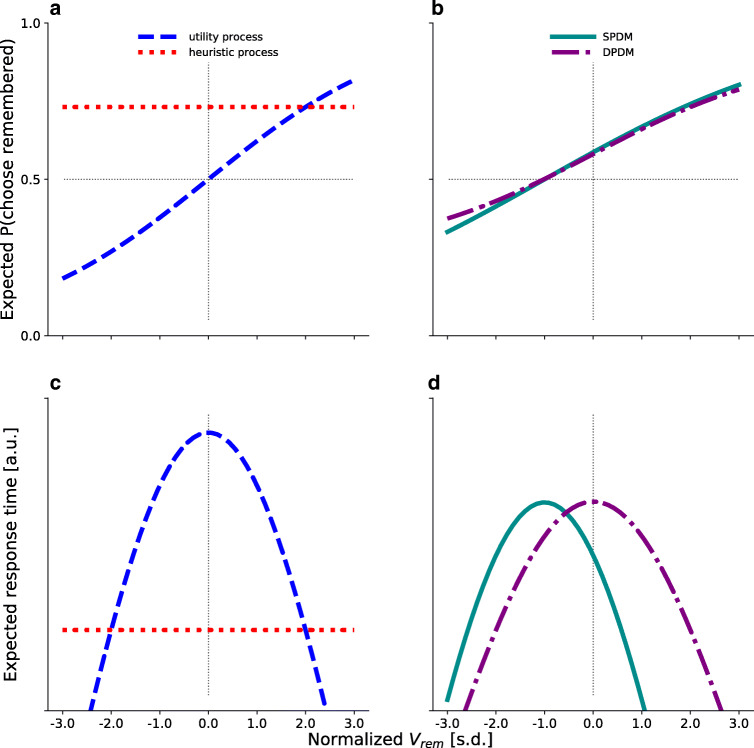
Qualitative predictions. **a** Expected choice curves of the utility process (*blue*, *dashed*) and the heuristic process (*red*, *dotted*) as a function of the value of the remembered snack *V*_*rem*_. **b** The weighted average of the two curves in **A** results in the expected choice curve of the DPDM (*purple*, *dashed-dotted*), which is depicted along with the expected curve for the SPDM (*cyan*, *solid*). **c** Expected RT curves for the utility and heuristic process. **d** Expected RT curves for the SPDM and DPDM

In the SPDM, the drift rate depends on *V*_*rem*_ and on the reference value *γ* (see Eq. 1), such that *ν* = 0 when *V*_*rem*_ = *γ*. Accordingly, the SPDM assumes participants to be indifferent between choice options whenever *V*_*rem*_ = *γ*. This is reflected in a sigmoidal choice-probability curve with its indifference point at a negative *V*_*rem*_ value (Fig. 3b, green, continuous line). Such a curve was shown to reflect the memory bias (Gluth et al., 2015) and was found in the choice data used in the present study (see Mechera-Ostrovsky & Gluth, 2018). Furthermore, the SPDM assumes that the RTs follow an inverted U-shape with its peak at a negative *V*_*rem*_ value (Fig. 3d, green, continuous line).

The DPDM assumes two drift rates of two independent diffusion processes, only one of which is selected in a given trial. On utility trials, individuals use a utility-maximizing strategy so that *ν*_*Utility*_ = *V*_*rem*_ − *V*_*avg*_, leading to *ν*_*Utility*_ = 0 when *V*_*rem*_ = *V*_*avg*_. This is illustrated in Fig. 3a (blue, dashed line), where the sigmoidal choice curve of the utility process has its indifference point at a *V*_*rem*_ = 0, which corresponds to the average value of all snacks. Accordingly, the RT curve follows an inverted U-shape with its maximum at *V*_*rem*_ = *V*_*avg*_ (Fig. 3c, blue, dashed line). In heuristic trials, the heuristic process accumulates evidence in favor of the remembered option, independently of its actual value. Since *ν*_*Heuristic*_ is independent of *V*_*rem*_, the choices and RTs are also independent of *V*_*rem*_ (Fig. 3a/c, red, dotted lines). The full predictions of the DPDM (Fig. 3b/d, purple, dash-dotted lines) will be a mixture of the predictions of its two sub-processes, depending on the mixture parameter Δ.

Crucially, the DPDM is virtually indistinguishable from the SPDM on the level of choices alone: The choice curves are shifted to the left, such that its indifference point is at a negative *V*_*rem*_ value. However, since the expected RT curve of the heuristic process is independent of *V*_*rem*_, the peak of the RT curve across all trials only depends on the utility process. Therefore, the RT curve of the DPDM follows an inverted U-shape with its peak expected RT at *V*_*rem*_ = *V*_*avg*_ (for a mathematical proof, see Appendix A2). In sum, while the SPDM and the DPDM cannot be distinguished based on the choice patterns, they can be distinguished based on their different predictions of RTs.

### Testing qualitative predictions

As outlined in the previous section, single- and dual-process accounts predict different RTs patterns. More specifically, the DPDM assumes that the RT curve peaks at *V*_*rem*_ = *V*_*avg*_, whereas the SPDM peaks at *V*_*rem*_ = *γ*, where *γ* < *V*_*avg*_. To test which of these assumptions is supported by the data, we fitted a quadratic function to the RT data:

5 $$ RT(V_{rem})={\upbeta}_{0}+{\upbeta}_{1}\times(V_{rem}+{\upbeta}_{2})^{2}  $$

This regression model describes a quadratic function with intercept β_0_ and slope β_1_. The term (*V*_*rem*_ + β_2_)^2^ shifts the maximum RT along the x-axis. If β_2_ equals zero, the curve is symmetrical around *V*_*avg*_ which matches the RT predictions of the DPDM. If β_2_ is larger than 0, the RT curve is shifted to the left, in line with the SPDM.

To estimate the β_2_ parameter, we fitted a hierarchical Bayesian regression model to the log-transformed and *z*-standardized RTs. RTs of every trial were predicted using $V_{rem_{t}}$, individual β parameters, and Gaussian noise. The individual-level β parameters (denoted by subscript *s*) were drawn from group-level normal distributions:

6 $$ \begin{array}{@{}rcl@{}} && {\upbeta}_{0,s} \sim {\mathcal{N}(\mu_{{\upbeta}_{0}}, \sigma_{{\upbeta}_{0}})}, \\ && {\upbeta}_{1,s} \sim {\mathcal{N}(\mu_{{\upbeta}_{1}}, \sigma_{{\upbeta}_{1}})}, \\ && {\upbeta}_{2,s} \sim {\mathcal{N}(\mu_{{\upbeta}_{2}}, \sigma_{{\upbeta}_{2}})}. \end{array} $$

At the group-level, all prior distributions were standard normal (for the *μ* parameters) and standard half-normal (for the *σ* parameters) distributions.

We leveraged the fact that, in case of the qualitative RT model, the DPDM’s prediction is nested within the SPDM’s prediction with the restriction $\mu _{{\upbeta }_2} = 0$. We applied a Gaussian kernel density estimation (with a bandwidth of .1) to the posterior samples of $\mu _{{\upbeta }_2}$ and obtained a Bayes factor using the Savage–Dickey density ratio test (e.g., Lee & Wagenmakers, 2013).

Importantly, the SPDM does not only predict a shift to the left in both the choice and the RT curves (see Fig. 3b and d), but it also assumes that these shifts arise from the same process. Therefore, these two shifts of choice and RT curves should be related to each other. To test this relationship, we quantified the *memory bias on choice* as the intercept parameter of a hierarchical logistic regression model. When this parameter is larger than 0, the choice curve shifts in favor of the remembered option. Analogously, we interpret the β_2_ parameter as *memory bias on response times*. To assess whether participants who exhibit a larger memory bias on choices also show a larger memory bias on RTs, we performed a Bayesian correlation analysis on the individual-level posterior medians of the intercept and ${\upbeta }_{2_s}$ parameters.

### Quantitative model comparison via hierarchical Bayesian modeling

In addition to comparing their qualitative predictions, we performed a quantitative model comparison using a hierarchical Bayesian approach. This comparison offers a more precise evaluation of the validity of the assumed cognitive processes. To the best of our knowledge, the DPDM (Alós-Ferrer, 2018) has only been used to derive qualitative predictions so far. Therefore, we consider our model comparison as providing a principled way of gauging the DPDM’s quantitative adequacy.

#### SPDM

In the SPDM, choice and RT data come from a diffusion process that results in a Wiener distribution:

7 $$ y \sim Wiener(a,z,T_{er},\nu). $$

In the hierarchical model, the subject-specific parameters (denoted by subscript *s*) are drawn from normal group-level distributions with respective group-level parameters *μ* and *σ* (hyper priors are listed in Appendix B1). Boundary separation *a*_*s*_, starting point bias *z*_*s*_ and non-decision time $T_{er_s}$ were estimated as

8 $$ \begin{array}{@{}rcl@{}} && a_{s} \sim e^{{\mathcal{N}(\mu_{a}, \sigma_{a})}}, \\ && z_{s} \sim {\Phi}({\mathcal{N}(\mu_{z}, \sigma_{z})}), \\ && T_{er_{s}} \sim e^{{\mathcal{N}(\mu_{T_{er}}, \sigma_{T_{er}})}}, \end{array} $$

where Φ denotes the cumulative distribution function of the standard normal. The drift rate parameter *ν* varies from trial to trial (subscript *t*) as follows:

9 $$ \begin{array}{@{}rcl@{}} \nu_{s,t} &=& d_{SPDM_{s}}[Rem_{right_{s,t}}\times(V_{right_{s,t}}-\gamma_{s}) - Rem_{left_{s,t}}\\&&\times(V_{left_{s,t}}-\gamma_{s})], \\ &&\!\!\!\!\!\!\!\!\!\!\!\!\!\!\!\!\!\! d_{SPDM_{s}} \sim e^{{\mathcal{N}(\mu_{d_{SPDM}}, \sigma_{d_{SPDM})}}}, \\ &&\!\!\!\!\!\!\!\!\!\!\!\!\!\!\!\!\!\! \gamma_{s} \sim {\mathcal{N}(\mu_{\gamma}, \sigma_{\gamma})}, \end{array} $$

where $Rem_{right_{s,t}}$ ($Rem_{left_{s,t}}$) are dummy variables, indicating whether the right (left) snack was remembered, and $V_{right_{s,t}}$ ($V_{left_{s,t}}$) indicating the subjective value of the right (left) snack. $d_{SPDM_s}$ is a free parameter which scales value differences to the speed of evidence accumulation. *γ* was the parameter which acts as reference value, indicating the biased value comparison in the SPDM.

#### DPDM

The DPDM is a mixture model, where the choices and RTs come from two different diffusion processes:

10 $$ \begin{array}{@{}rcl@{}} && y \sim {\Delta}\times Wiener(a,z,T_{er},\nu_{Utility}) + (1-{\Delta})\\&&\times Wiener(a,z,T_{er},\nu_{Heuristic}), \\ && {\Delta}_{s} \sim {\Phi}({\mathcal{N}(\mu_{\Delta}, \sigma_{\Delta})}). \end{array} $$

The mixing parameter Δ indicates the proportion of trials in which the response was generated by the utility process.

While both processes share the same parameters *a*, *z* and *T*_*er*_, they differ with respect to their drift rates *ν*_*Utility*_ and *ν*_*Heuristic*_. *ν*_*Utility*_ is the same as in the SPDM but compares *V*_*rem*_ to the average snack value ($V_{avg_{s}}$) instead of *γ*.

11 $$ \begin{array}{@{}rcl@{}} \nu_{Utility_{s,t}} &=& d_{Utility_{s}}[Rem_{right_{s,t}}\times(V_{right_{s,t}}-V_{avg_{s}})\\ &&- Rem_{left_{s,t}}\times(V_{left_{s,t}}-V_{avg_{s}})], \\ && d_{Utility_{s}} \sim e^{{\mathcal{N}(\mu_{d_{Utility}}, \sigma_{d_{Utility}}})}. \end{array} $$

The drift rate of the heuristic process is a free parameter:

12 $$ \begin{array}{@{}rcl@{}} && \nu_{Heuristic_{s,t}} = d_{Heuristic_{s}}[Rem_{right_{s,t}} - Rem_{left_{s,t}}], \\ && d_{Heuristic_{s}} \sim e^{{\mathcal{N}(\mu_{d_{Heuristic}}, \sigma_{d_{Heuristic}}}}. \end{array} $$

Note that the exponential transformation enforces a positive drift rate in the direction of the remembered option.

#### Model fitting and model comparison

We estimated the parameters of both hierarchical models with *Stan* (Stan-Development-Team, 2018) using a No-U-Turn sampler (Hoffman & Gelman, 2014). Each model was estimated with four chains of 10,000 iterations each (50*%* of which were warm-up iterations that were discarded). To ensure model convergence using the $\hat {R}$ statistic (Gelman & Rubin, 1992), we checked that $\hat {R} <= 1.01$ for all parameters. We compared the penalized-for-complexity fit of both models using the widely applicable information criterion (WAIC; Watanabe, 2013).

As all model comparison procedures are relative measures of fit (i.e., they can only assess the model performance relative to other models), we generated posterior predictive distributions for choice rates and RTs as a function of *V*_*rem*_ to evaluate absolute model performance (i.e., the degree to which they are able to capture quantitative and qualitative patterns in the empirical data). To do so, we simulated 500 experiments using virtual agents who behaved according to the model equations. Note that in the DPDM simulations, each trial was either a heuristic or utility trial, determined by the outcome of a Bernoulli trial with probability Δ_*s*_. Each agent’s parameter vector was drawn randomly from the posterior distribution obtained during model fitting. We aggregated the choices and RTs across trials and participants into 8 bins and calculated the respective 95% highest-density interval (HDI; Kruschke, 2015). Using parameter- and model-recovery analyses we confirmed that both models were able to recover data-generating parameters and that the models make different predictions with respect to behavior such that a data-generating model can be correctly identified (see Appendix B2 for details).

## Results

### Qualitative results

**Fig. 4:**
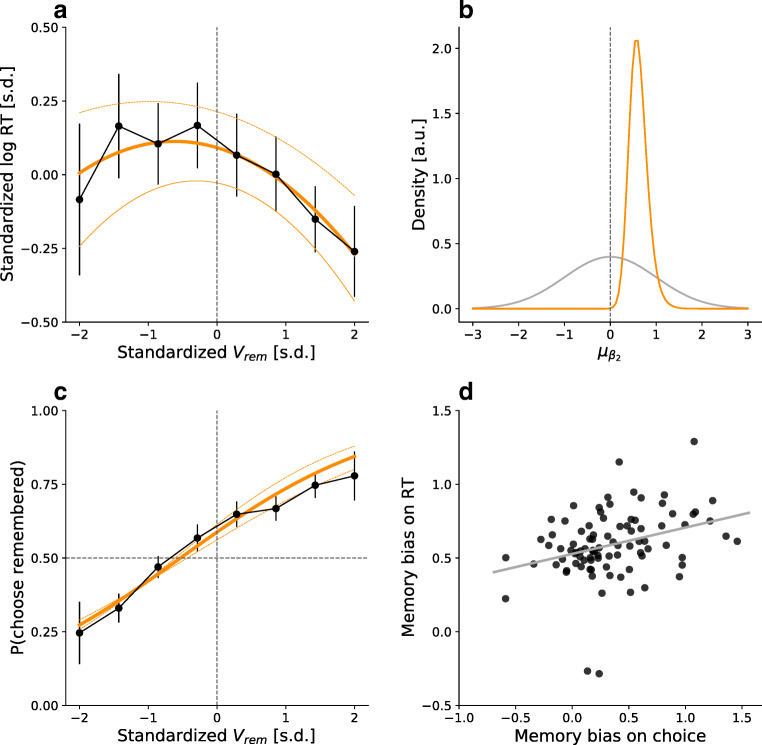
Qualitative results. **a** Average RTs depending on *V*_*rem*_ in *black*. *Error bars* depict the empirical 95% CIs. The *orange curve* yields the predicted RT curves from the RT Model, using the posterior mean. The *dotted curves* indicate the predicted RTs of a 95% highest density interval of the posterior distributions. **b** Prior (*grey*) and posterior (*orange*) parameter distributions of *μ*_β2_. **c** Empirical choices (*black*) and the estimated choice curve (*orange*). **d** Correlation of the individual estimates of memory bias on choice, and the memory bias on RT

Our first approach to compare a single- and a dual-process account of memory-based decisions making was to evaluate the qualitative predictions of the respective approaches by fitting a regression model (5) to the RT data. As outlined in the Method section, the single-process account predicts that the RT curve follows an inverted U-shape (as a function of value difference) with its peak at a negative value of *V*_*rem*_. This shift is quantified by the parameter $\mu _{{\upbeta }_2}$ (see Eq. 5). In line with this prediction, the posterior distribution of $\mu _{{\upbeta }_2}$ was positive and the 95% HDI excluded zero (*M* = .62, 95% HDI: [.30,.98]). The individual-level means were distributed around the posterior $\mu _{{\upbeta }_2}$ with a $\sigma _{{\upbeta }_2}$ (*M* = .49, 95% HDI: [.17,.86]). The RT model predicted the empirical RT data very well (Fig. 4a). To directly compare the predictions of the SPDM and the DPDM, we obtained a Bayes factor using the Savage–Dickey density ratio that tested whether $\mu _{{\upbeta }_2}$ is different from 0. We obtained very strong evidence in favor of the alternative hypothesis that $\mu _{{\upbeta }_2}$ is not 0, with a Bayes factor of 128.9, providing further support for the SPDM model.

Because the single-process account predicts that the memory bias on RT and the memory bias on choice arise from the same underlying process, we also fitted a hierarchical logistic regression model to the individual choice data. The group-level intercept indicated a shift of the choice curve in line with the memory bias on choice (*M* = .35, 95% HDI: [.25,.46]) and reproduces the non-Bayesian results reported by Mechera-Ostrovsky and Gluth (2018). Figure 4c depicts the predicted choice curve as a function of *V*_*rem*_.

We correlated the medians of the participant level intercept parameter distributions (which reflect the memory bias on choice of each participant) with the β_2*s*_ parameters of the RT-model (which reflect the memory bias on RT). As predicted, the individual intercepts from the regression model correlated positively with the β_2*s*_ estimates from the RT-Model (*r* = .315, 95% HDI: [.12,.49], Fig. 4d). Thus, the shift in the RT curve is associated with the shift in the choice curve, suggesting a common underlying mechanism.

### Quantitative results

In addition to the qualitative analysis, we compared the SPDM and the DPDM on a quantitative level by a model comparison within a hierarchical Bayesian framework. Summary statistics of the posterior distributions of all group-level parameters are provided in Table 1.

**Table 1:** Group-level parameter estimates for the single- and dual-process diffusion models.

Model	Parameter	*M*	95% HDI
SPDM	*μ*_*a*_	1.77	[1.68, 1.88]
	*σ*_*a*_	1.30	[1.25, 1.35]
	$\mu _{T_{er}}$	0.29	[0.27, 0.32]
	$\sigma _{T_{er}}$	1.46	[1.38, 1.57]
	*μ*_*z*_	0.52	[0.51, 0.52]
	*σ*_*z*_	1.05	[1.03, 1.08]
	$\mu _{d_{SPDM}}$	0.36	[0.31, 0.41]
	$\sigma _{d_{SPDM}}$	1.70	[1.51, 1.93]
	*μ*_*γ*_	− 0.54	[− 0.70,− 0.39]
	*σ*_*γ*_	1.82	[1.58, 2.10]
DPDM	*μ*_*a*_	1.82	[1.73, 1.93]
	*σ*_*a*_	1.31	[1.26, 1.36]
	$\mu _{T_{er}}$	0.29	[0.26, 0.31]
	$\sigma _{T_{er}}$	1.48	[1.38, 1.57]
	*μ*_*z*_	0.52	[0.51, 0.52]
	*σ*_*z*_	1.06	[1.04, 1.09]
	$\mu _{d_{Utility}}$	0.70	[0.55, 0.85]
	$\sigma _{d_{Utility}}$	1.54	[1.30, 1.86]
	$\mu _{d_{Heuristic}}$	0.29	[0.17, 0.48]
	$\sigma _{d_{Heuristic}}$	3.03	[2.03, 4.62]
	*μ*_Δ_	0.55	[0.44, 0.66]
	*σ*_Δ_	1.84	[1.49, 2.32]

Note. *M* represents the posterior mean. The 95%-HDI depicts the boundaries of the 95% highest-density interval. Note that these values are transformed with respective transformation functions (see main text)

We relied on the WAIC for model comparison. The SPDM had a lower WAIC than the DPDM (17,394 vs. 17,468), with a difference in WAICs of 74.34 (*SE* = 24.74), resulting in a strong standardized effect size of $\frac {\Delta \text {WAIC}}{SE} = 3.01$.

**Fig. 5:**
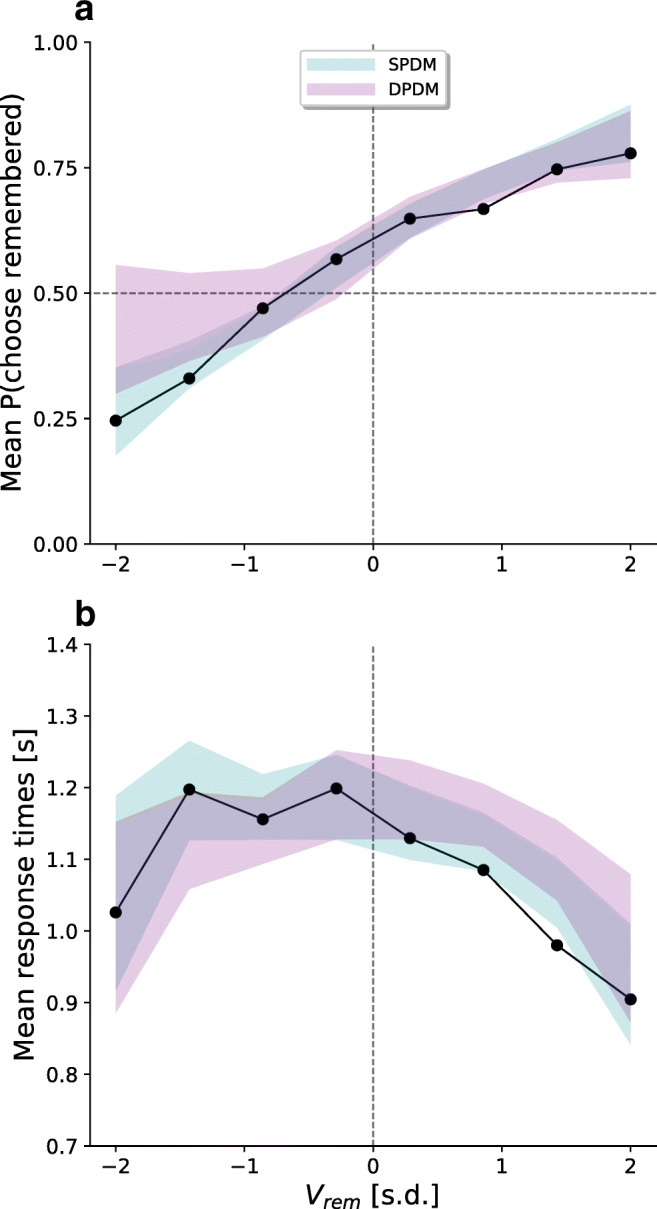
Posterior predictives. **a** shows the 95% HDI of the means of simulated data sets, based on the estimated posterior parameter distributions of the SPDM (*cyan*) and DPDM (*purple*). The *black dots* indicate the empirical means. **b** shows the same for response times

We performed posterior predictive checks to assess absolute model performance. Both models are capable to produce a shift in the choice curve (see Fig. 5a). However, whereas the SPDM predicts most data points well (i.e., within the 95% HDI), the empirical data often lie outside the 95% HDI of the DPDM, particularly when *V*_*rem*_ is low. With respect to RTs, this difference is even stronger (Fig. 5b). Specifically, when *V*_*rem*_ was below average, the DPDM underestimated the RTs. When it was above average, it overestimated them. This result is in line with the notion that the DPDM is unable to account for a shifted U-shaped RT curve, as it is forced to predict a curve that is symmetrical around zero. In contrast, the SPDM provides an accurate account of the empirical RT curve. Taken together, both the relative and the absolute model comparisons confirm the qualitative results and provide additional support for a single- and against a dual-process account of memory-based decisions.

## Discussion

The present study compared a single- with a dual-process account of memory-based decisions. The single-process account assumes that memory affects the valuation of options, such that better-remembered options are perceived as more valuable. In contrast, the dual-process account assumes that each decision is made by one of two processes, where a rational process competes with a heuristic-based process which ignores value and uses memory-strength information only. While the single-process account was already tested before (Gluth et al., 2015), the dual-process account, implemented on the basis of the DPDM as proposed by Alós-Ferrer (2018), has never been tested in the current context. This study thus provides a first empirical test of these opposing theories on memory-based decisions. We found that both models can make similar predictions with respect to choices but differ regarding their predictions of RTs. Using previously published data from a memory-based choice task (Mechera-Ostrovsky and Gluth, 2018), we found consistent support for the single-process account in both qualitative and quantitative analyses.

Our results bear strong analogies to a debate on the use of the recognition heuristic in inference tasks. The recognition heuristic states that people judge recognized items as being more important / frequent / larger than unrecognized items (Goldstein & Gigerenzer, 2002). Originally, the heuristic was not conceptualized in the framework of dual-process accounts. However, to account for the fact that people do not always go with the recognized cue, it has been argued that the heuristic is applied in some but not all trials (Pachur, 2011). Yet, this proposal was refuted by a recent study that–similar to our approach–relied on RT data to dissociate between the recognition heuristic and competing theories of inferential judgements (Heck & Erdfelder, 2017).

Despite being very popular in judgment and decision-making and other psychological disciplines (Evans, 2008), dual-process theories often became a target of fundamental criticism for conceptual issues but also for the lack of empirical support (Keren & Schul, 2009; Kruglanski & Gigerenzer, 2011; Melnikoff & Bargh, 2018). One shortcoming of many dual-process theories is their poor formalization which impedes quantitative model comparison (Diederich & Trueblood, 2018). This is especially true, when between-trial dynamics which account for choice and RT differences are ignored (Krajbich et al., 2015). In this study, we outlined a principled way to test a dual-process against a single-process account by means of quantitative and qualitative model comparison. Our approach is based on a recently developed formal model of a dual-process account (Alós-Ferrer, 2018) with suitable assumptions for testing our particular hypothesis. Apart from model comparison, estimation of the model also offers a deeper understanding of the underlying processes, such as the relative proportion of the two presumed processes (e.g., Δ), or their within-trial dynamics (e.g., drift rates). We believe the field of judgment and decision-making is well advised to formalize the proposed dual-process models and to test their empirical content. This approach has the potential to move the debate on dual-process models forward by adhering to empirical findings and methodological rigor.

Within the dual-process framework, there are two types of conceptualization which specify how the two processes can be implemented (Evans, 2008). According to the *parallel-competitive* structure, type 1 and type 2 processes run in parallel and a potential conflict between them has to be resolved to determine which process is applied in a given decision. The DPDM of Alós-Ferrer (2018) can be assigned to this group of models. The second influential dual-process architecture comprises the *default-interventionist* models. These models assume that a type 1 process is activated to generate an intuitive default response but may be overcome by the reflective type 2 process. From a diffusion model perspective, such a process could be reflected in a starting point bias in favor of the intuitive option (e.g., Chen and Krajbich, 2018). Hence, we also tested whether the assumption of a starting point bias towards the remembered option could account for the present data, but found that it cannot (see Appendix C1). An alternative implementation of a default-interventionist model was proposed by Diederich and Trueblood (2018), who investigated risky choices, drawing on prospect theory (Kahneman & Tversky, 1979) and expected utility theory (Neumann & Morgenstern, 1953). In our study, however, we were interested in value-based decisions, which are directly covered by the DPDM of Alós-Ferrer (2018) but not by Diederich and Trueblood (2018).

The scientific process of model selection is based on the principle of parsimony (Occam’s razor). According to this principle, we should prefer hypotheses that can account for a complex phenomenon drawing on a few (rather than many) assumptions. In this study, we found empirical support that a single decision process provides a more parsimonious explanation of memory-based choices compared to a dual-process account. This parsimony is supported both by the Bayes factors and information criteria analyses, established methods to compare models in terms of fit and parsimony (Vandekerckhove et al., 2015). On a more conceptual level, the single-process account could be said to draw on fewer assumptions than the dual-process account. On the one hand, the single-process account can account for the data assuming a single comparison process, between remembered option and a biased reference value. On the other hand, the dual-process account assumes a computationally demanding utility maximizing process, a heuristic process depending on recognition of an item, and a central executive which selects among these processes.

In sum, our results clearly indicate that a single decision process in which the evaluation process is biased by memory describes the memory bias better than a dual-process account that assumed two independent processes (i.e., a memory-heuristic and a utility process). We fit both models in a hierarchical Bayesian modeling framework and outlined a rigorous procedure to empirically test single- and dual-process accounts of decision-making.

## Figures and Tables

**Fig. 6:**
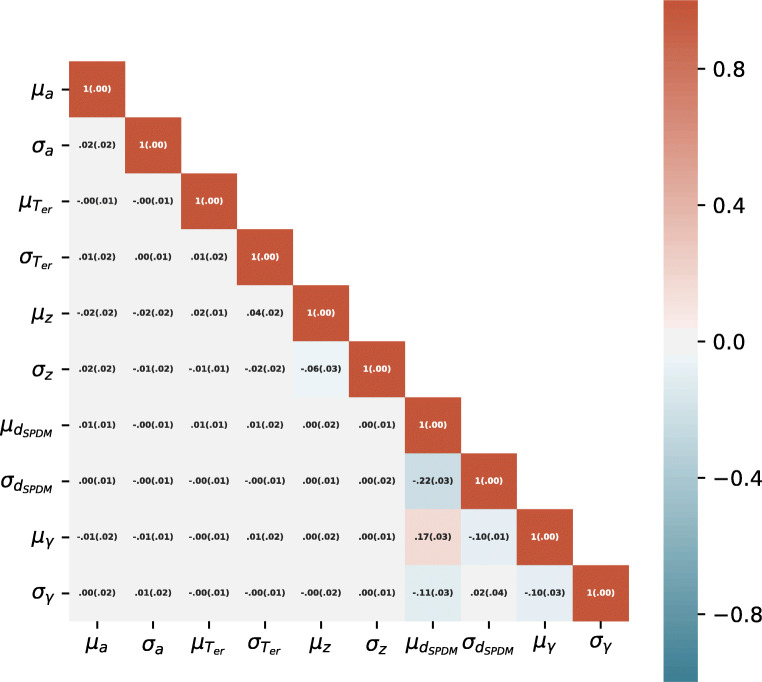
Correlations of group parameter posterior samples of the SPDM. Each cell shows the mean correlation coefficients (using Fisher’s transformation) with the corresponding standard deviation in brackets

**Fig. 7:**
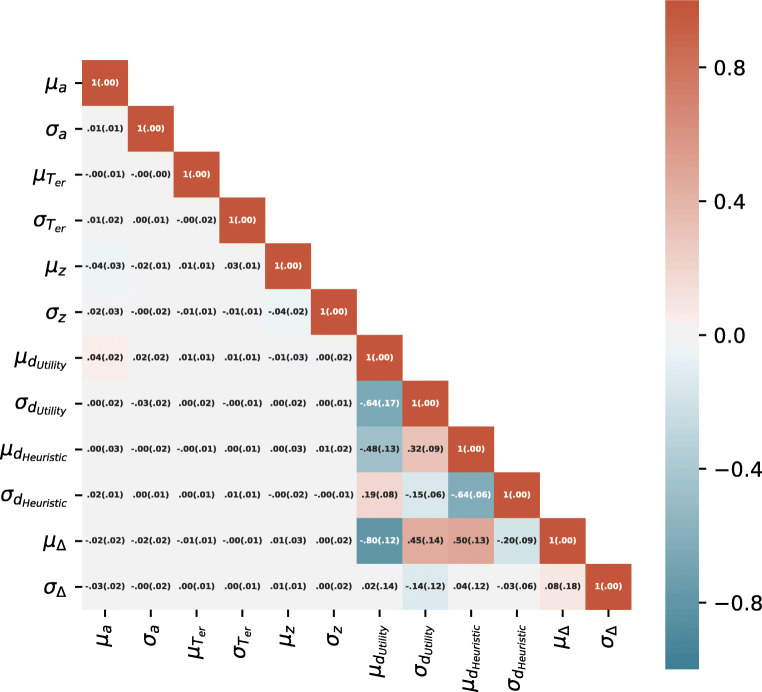
Correlations of group parameter posterior samples of the DPDM. Each cell shows the mean correlation coefficients (using Fisher’s transformation) with the corresponding standard deviation in brackets

**Fig. 8:**
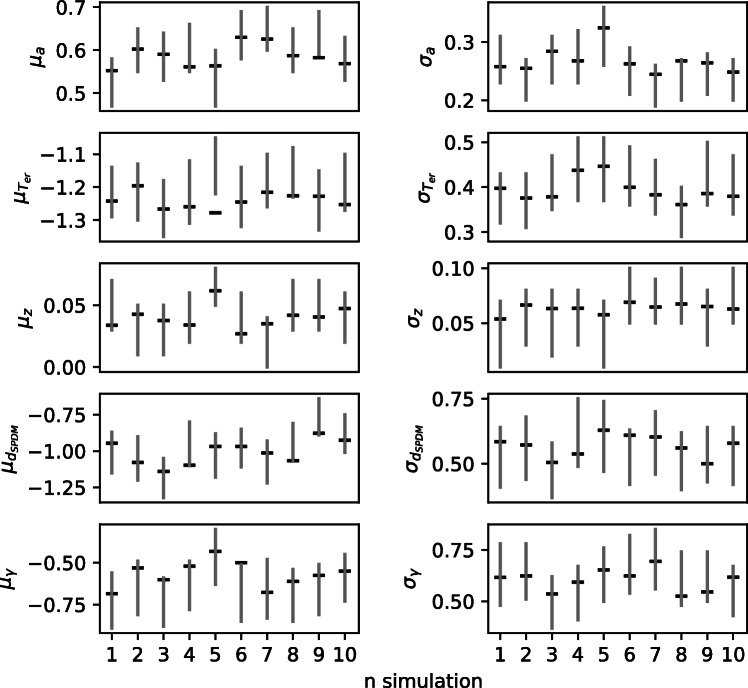
Identifiability of the SPDM group parameters. *Horizontal black lines* indicate the generating parameter values for the ten simulations. *Vertical lines* indicate the 95% HDI of the estimated parameter distributions

**Fig. 9:**
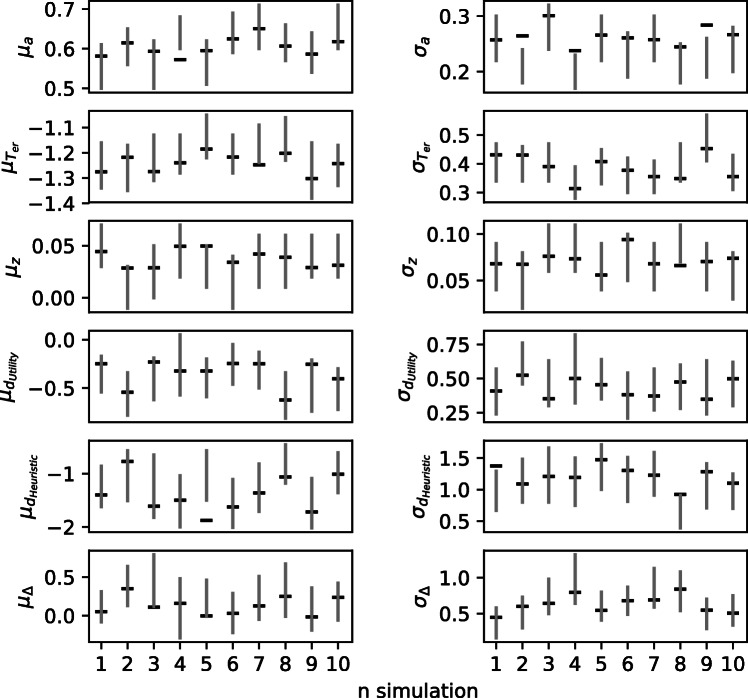
Identifiability of the DPDM group parameters. *Horizontal black lines* indicate the generating parameter values for the ten simulations. *Vertical lines* indicate the 95% HDI of the estimated parameter distributions

**Fig. 10:**
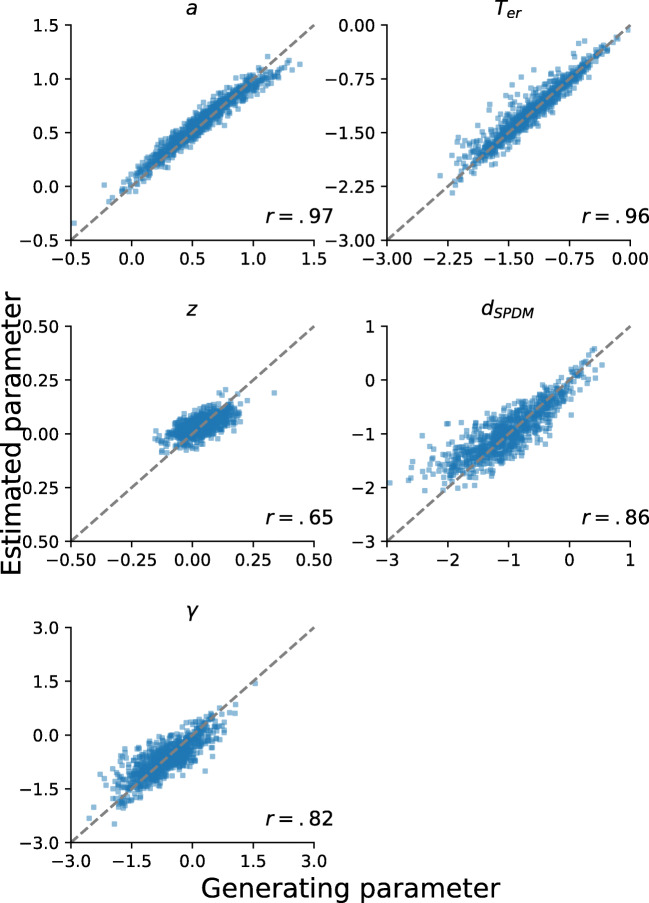
Identifiability of the SPDM individual level parameters, pooled over all ten simulations. The position on the *X*-axes indicate generating parameter value, the *Y* -position show the estimated posterior mean. Values around the *dashed grey identity line* yield good recovery. The Pearson’s correlation coefficients of generated and estimated parameters are indicated on the lower left for each parameter

**Fig. 11:**
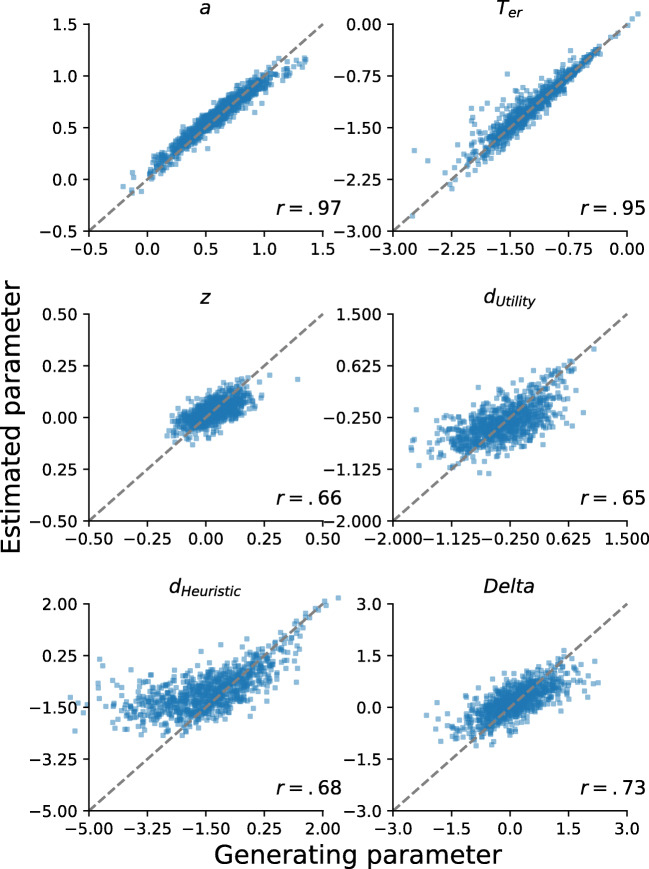
Identifiability of the DPDM individual level parameters, pooled over all ten simulations. The position on the *X*-axes indicate generating parameter value, the *Y* -position show the estimated posterior mean. Values around the *dashed grey identity line* yield good recovery. The Pearson’s correlation coefficients of generated and estimated parameters is indicated on the lower left for each parameter

**Fig. 12:**
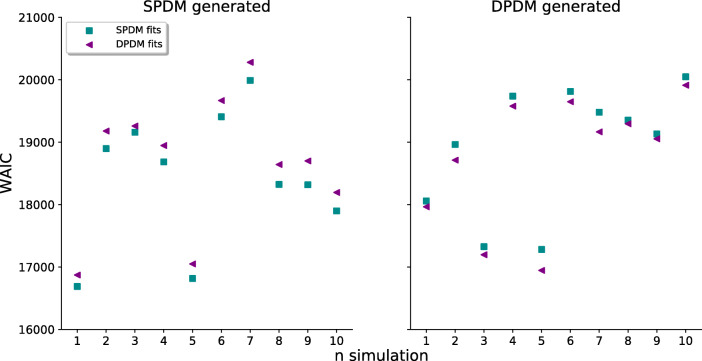
Model recovery. The plot shows the WAIC values for the parameter fits of the SPDM (*cyan rectangles*), and the DPDM (*purple triangles*) for each simulated data set. The *left panel* shows the datasets generated from the SPDM, the *right panel* shows those from the DPDMx

**Fig. 13:**
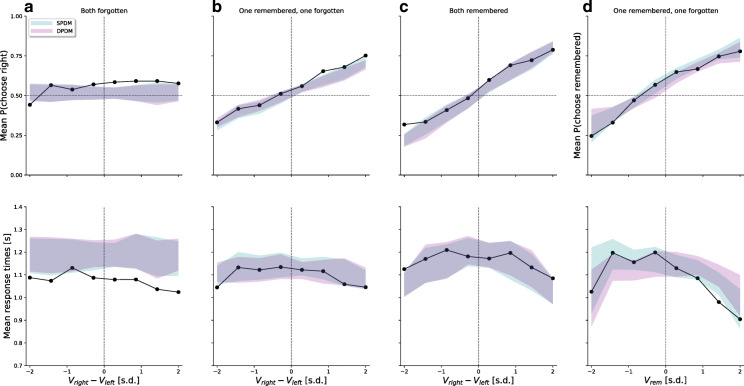
Posterior simulations for all trial types. The *black dots* indicate the empirical means for choices (*upper panels*) and response times (*lower panels*). The *colored areas* indicate the 95% HDI of the means of simulated data sets based on the estimated posterior parameter distributions of the SPDM (*cyan*) and DPDM (*purple*). **a**, **b**, and **c** indicate the posterior simulations for “both forgotten”, “one remembered, one forgotten”, and “both remembered” trials, respectively. Note that these simulations depend on the value differences between the right and the left snack items, and the choices indicate the mean proportions of choices that favored the right option. **d** shows the posterior simulations for “one remembered, one forgotten” trials as a function of *V*_*rem*_ analogously to Fig. 5

## Appendix

### A1 Trial types

In the remember-and-decide task, there are three types of trials, based on the number of snacks remembered (i.e., both remembered; both forgotten; one remembered, one forgotten). For this study, only the last trial type was of interest, as the memory bias (i.e., the tendency to prefer remembered over forgotten options) can only be tested with these kinds of trials. Table 2 summarizes the trial numbers for each trial type.

**Table 2:** Numbers of different trial types

Trial type	Total	*M*	*SD*	Range
Both forgotten	5651	62.8	32.9	13-163
One remembered, one forgotten	8031	89.2	15.8	39-118
Both remembered	5468	60.8	30.0	7-142

Note. Total: Number of trials in the data set; *M*: mean trial number per participant; *SD*: standard deviation; Range: range of trials between participants

### A2 Analytical derivation of expected mean response times

In the main text, we illustrate the RT predictions of the DPDM (and the SPDM) using simulations. Predictions for the expected RT (and for the symmetry of the predicted RT curve around 0; see Fig. 1) can also be derived analytically. Here, we report these derivations.

The DPDM describes the expected RTs (eRT) as the weighted sum of mean RTs of the heuristic and the utility process. It can be described as

13 $$ \begin{array}{@{}rcl@{}} eRT_{\Delta V} &= & p(H)\times p(R|H)\times eRT(R|H) + p(H)\\ &&\times[1-p(R|H)]\times eRT(F|H) + [1-p(H)] \\ &&\times p(R|U,{\Delta} V,\gamma)\times eRT(R|U,{\Delta} V,\gamma)\\ && + [1-p(H)]\times [1-p(R|U,{\Delta} V,\gamma)]\\ &&\times eRT(F|U,{\Delta} V,\gamma) \end{array} $$

where *e**R**T*_Δ*V*_ is the expected RT for the value difference Δ*V*, *p*(*H*) is the probability of the heuristic process, *p*(*R*) [*p*(*F*)] is the probability of remembering [forgetting] a snack, and *e**R**T*(*R*) [*e**R**T*(*F*)] is the mean RT for remembered [forgotten] snacks. The probability of a utility process is 1 − *p*(*H*), the probability and mean RT of remembered and forgotten choices in utility trials depend on Δ*V* and the reference value *γ* which is equal to *V*_*a**v**g*_ in the DPDM. Since we assume that the RTs come from a diffusion process, such that *e**R**T*(*c**h**o**o**s**e**R*) = *e**R**T*(*c**h**o**o**s**e**F*), Eq. 13 can be simplified to

14 $$ \begin{array}{@{}rcl@{}} eRT_{\Delta V} &=& p(H)\times eRT(R|H) + [1-p(H)]\\ &&\times eRT(R|U,{\Delta} V,\gamma). \end{array} $$

Because the utility process is the only one that depends on *V*_*r**e**m*_ and assumes the lowest drift rate when *V*_*r**e**m*_ = *V*_*a**v**g*_, the predicted peak of the RT is at *V*_*a**v**g*_. The same logic and equation can be applied to the SPDM by setting *p*(*H*) to 0 and allowing the reference value *γ* to be a free parameter. Then, the predicted peak RT in the single-process account is at *γ*.

### B1 Prior distributions of group parameters

In our hierarchical models, the individual model parameters were all drawn from normal distributions at the group level. Thus, for each model parameter, we estimated a group mean and a group standard deviation, on top of the individual parameters themselves. All group means were given a weakly-informative normal prior, and all group standard deviations were given a weakly-informative half-normal prior. The prior distributions for the group-level parameters are listed in Table 3.

**Table 3:** Prior distributions for group parameters.

Group parameter	SPDM	DPDM
*μ*_*a*_	$\mathcal {N}(0,3)$	$\mathcal {N}(0,3)$
*σ*_*a*_	$\mathcal {HN}(0,3)$	$\mathcal {HN}(0,3)$
*μ*_*z*_	$\mathcal {N}(0,1)$	$\mathcal {N}(0,1)$
*σ*_*z*_	$\mathcal {HN}(0,1)$	$\mathcal {HN}(0,1)$
$\mu _{T_{er}}$	$\mathcal {N}(-1,1)$	$\mathcal {N}(-1,1)$
$\sigma _{T_{er}}$	$\mathcal {HN}(0,3)$	$\mathcal {HN}(0,3)$
$\mu _{d_{SPDM}}$	$\mathcal {N}(0,3)$	−
$\sigma _{d_{SPDM}}$	$\mathcal {HN}(0,3)$	−
*μ*_*γ*_	$\mathcal {N}(0,1)$	−
*σ*_*γ*_	$\mathcal {HN}(0,3)$	−
$\mu _{d_{Utility}}$	−	$\mathcal {N}(0,3)$
$\sigma _{d_{Utility}}$	−	$\mathcal {HN}(0,2)$
$\mu _{d_{Heuristic}}$	−	$\mathcal {N}(0,3)$
$\sigma _{d_{Heuristic}}$	−	$\mathcal {HN}(0,2)$
*μ*_Δ_	−	$\mathcal {N}(0,3)$
*σ*_Δ_	−	$\mathcal {HN}(0,3)$

### B2 Parameter and model recovery analyses

#### Parameter recovery

We performed a parameter-recovery analysis for both models. For each model, we chose the ten group-level parameter estimates from the respective models’ posteriors that had the highest log-likelihood. From these group-level distributions, we drew 90 independent samples that determined individual-level behavior of the artificial agents. As inputs for the agents, we used the same inputs that we used for model fitting. For the DPDM, each trial was simulated to be either a heuristic or utility trial, based on independent Bernoulli trials ${\mathscr{B}}({\Delta }_s)$. We re-fitted each of these generated datasets with the respective models to assess the degree to which parameters could be recovered.

Following an analysis approach implemented in Fontanesi et al., (2019), we addressed three questions in this analysis: 1) Do parameter values trade off during the estimation procedure (i.e., to which degree do models suffer from sloppiness)? 2) Are the models able to correctly identify the group-level parameters? 3) Are the models able to correctly identify the individual-level parameters?

To address question 1, we calculated Pearson correlation coefficients between the posterior samples of all group parameters of each of the ten fitted artificial data sets. Subsequently, the ten correlation coefficients were Fisher-transformed and subsequently averaged and their standard distribution was calculated. Note that group parameters were drawn independently from each other. Therefore, there should be no correlation between the posterior samples. Figure 6 shows the mean correlations between the parameters. Overall, we only observed weak correlations between *μ*_*d**S**P**D**M*_ and *σ*_*d**S**P**D**M*_, and *μ*_*d**S**P**D**M*_ and *μ*_*γ*_. The DPDM suffered from stronger sloppiness, especially between $\mu _{d_{Utility}}$, $\mu _{d_{Heuristic}}$ and *μ*_Δ_ (Fig. 7).

To address question 2, we calculated the 95% HDIs of the posterior group-level parameter distributions. If the parameters are identifiable, the 95% HDI of should include the true data-generating parameter value (see also Spektor and Kellen, 2018). We observed good identifiability of the group-level parameters for both models (Figs. 8, 9, 10 and 11).

To address question 3, we correlated the true data-generating parameters on the individual level with the estimated means of the posterior distributions. For this analysis, the means were transformed back to the space of the group-level parameters. For both models, we observed very good recovery of the *a* and *T*_*e**r*_ parameters. The recoverability of *z* was lower but still fair which may be due to the low explanatory power of this parameter. The parameters which specified the drift rate were recovered well for the SPDM, and fairly well for the DPDM. Overall, we judge the recoverability to be good for the SPDM and relatively good for the DPDM, and sufficient for both models.

#### Model recovery

We performed a model-recovery analysis to assess whether both models could in principle be identified as the winning model, given that the data are generating by the respective model. From the parameter recovery analysis, we used the 20 simulated set of data of which ten were generated by each of the models. We fitted both models to each set of data separately and calculated the WAICs of the fits. The critical test for the model recovery was whether a model from which the data was generated wins the comparison against the competing model on that data. In other words, given the data were generated with the SPDM, the WAIC of the SPDM should be lower than the WAIC of the DPDM (and vice versa for the DPDM). The SPDM outperformed the DPDM on data which was generated under the SPDM (Fig. 12). The DPDM, on the other hand, won the model comparison against the SPDM in all sets of data that came from the DPDM, showing good model recovery.

### B3 Analyses on all trial types

The main text focused on trials in which participants remembered one option but forgot the other one. These are the only trials in which the memory bias has an influence on participants’ behavior. In trials in which both options are remembered, the SPDM assumes a purely utility-driven choice process (see Eq. 9) because the bias parameter *γ*_*s*_ is cancelled out from the drift rate *ν*_*s*,*t*_. When both options are remembered, the DPDM would also solely rely on the utility process, since the assumed recognition heuristic is not applicable. In trials in which both options are forgotten, the drift rate of both the SPDM and the DPDM equals zero (again, the DPDM’s heuristic process is not applicable). In sum, both models make the same predictions for trials in which no option and both options are remembered.

Nonetheless, we fitted both models to data from all three trial types to check whether our results would still hold. Apart from including all trials, we followed the same procedures as in the main text. The posterior parameter distributions are summarized in Table 4. For the SPDM, the parameters were similar to those reported in the main text. On the other hand, the parameters affecting the drift rate in the DPDM differed when fitting the model to all trial types. $mu_{d_{Utility}}$ was estimated to be lower, $mu_{d_{Heuristic}}$ was estimated to be higher, and Δ increased substantially, indicating a higher proportion of utility trials.

**Table 4:** Group-level parameter estimates for the single- and dual-process diffusion models, considering all trial types

Model	Parameter	*M*	95% HDI
SPDM	*μ*_*a*_	1.80	[1.70, 1.90]
	*σ*_*a*_	1.28	[1.25, 1.34]
	$\mu _{T_{er}}$	0.26	[0.24, 0.29]
	$\sigma _{T_{er}}$	1.51	[1.42, 1.62]
	*μ*_*z*_	.52	[.51,.52]
	*σ*_*z*_	1.05	[1.03, 1.06]
	$\mu _{d_{SPDM}}$	0.31	[0.27, 0.35]
	$\sigma _{d_{SPDM}}$	1.70	[1.52, 1.92]
	*μ*_*γ*_	− 0.62	[− 0.79,− 0.44]
	*σ*_*γ*_	1.97	[1.70, 2.34]
DPDM	*μ*_*a*_	1.80	[1.72, 1.90]
	*σ*_*a*_	1.28	[1.25, 1.34]
	$\mu _{T_{er}}$	0.26	[0.24, 0.28]
	$\sigma _{T_{er}}$	1.51	[1.40, 1.60]
	*μ*_*z*_	.52	[.51,.52]
	*σ*_*z*_	1.05	[1.04, 1.07]
	$\mu _{d_{Utility}}$	0.32	[0.27, 0.36]
	$\sigma _{d_{Utility}}$	1.75	[1.57, 1.99]
	$\mu _{d_{Heuristic}}$	1.00	[0.84, 1.16]
	$\sigma _{d_{Heuristic}}$	1.21	[1.00, 1.42]
	*μ*_Δ_	.93	[.84,.97]
	*σ*_Δ_	3.60	[2.41, 5.70]

Note. *M* = posterior mean. HDI = highest-density interval. Note that these values are reported after applying their respective transformations (see main text)

As in the main text, quantitative model comparison favored the SPDM (WAIC = 44,465) over the DPDM (WAIC = 44,645) with a difference in WAICs of 179.98 and a standardized effect size of $\frac {\Delta \text {WAIC}}{SE} = 5.97$.

The posterior predictives yielded very similar predictions for both models (see Fig. 13a–c). Notably, while both models accounted relatively well for the “one remembered, one forgotten” and “both remembered” trial types, they made inaccurate predictions for the “both forgotten” trials. This results from the drift rate being zero in the “both forgotten” trials, which implies the prediction of slow RTs and random choices. In contrast to these predictions, the observed choices yielded a slight tendency to choose the right option (explained by a starting point bias *μ*_*z*_ > .5) and the RTs were not slower than those of the other trial types. We would argue that participants may have simply guessed, when they knew that they could not remember either of the two options.

In sum, both models can be fitted and account all trial types apart from the “both forgotten” trials. In line with the results reported in the main text, the SPDM provides a better account of the data than the DPDM. Moreover, the parameter estimates of the SPDM remain stable, whereas those of the DPDM change substantially, reflecting the robustness of the SPDM, and the rather low robustness of the DPDM.

### C1 Does a default-interventionist process explain the memory bias?

Apart from the parallel-competitive dual-process account of Alós-Ferrer (2018), it is conceivable that a default-interventionist account could explain the memory bias. Such an approach assumes that during each decision, rapid pre-conscious processes either approve of the type 1 decision, or intervene by initiating the rational type 2 process (Evans, 2008). From a diffusion model perspective, a default-interventionist account resembles a biased starting point *z* (Chen and Krajbich, 2018). More specifically, assuming that the default response would be implemented as a starting-point bias, the diffusion process would be biased so that the intuitive option would be selected quicker and more often than the non-intuitive one. Technically, this would still be a single-process account because the default option cannot finalize the decision process (there is some deliberation necessary), but its predictions resemble a default-interventionist dual-process model (e.g., fast errors).

With respect to the remember-and-decide task, this means that the starting point bias should favor the remembered option. If a biased starting point provided a better explanation for the memory bias, the memory bias parameter should be affected when a model is estimated with a free starting point which can favor the remembered option.

To evaluate this, we fitted another diffusion model to the data. We refer to this model as ”memory-bias-as-starting-point-bias model”. In order to fit the starting-point bias parameter, we reparameterized the DDM so that the upper boundary would represent choices in favor of the remembered option, and the lower boundary would represent choices against it. Other than that, the model was parameterized similarly to the SPDM (see Eq. 8)

15 $$ \begin{array}{@{}rcl@{}} && \nu_{s,t} = d_{s}(V_{rem_{s,t}}-\gamma_{s}), \\ && d_{s} \sim e^{{\mathcal{N}(\mu_{d_{SPDM}}, \sigma_{d_{SPDM})}}}, \\ && \gamma_{s} \sim {\mathcal{N}(\mu_{\gamma}, \sigma_{\gamma})}, \end{array} $$

where $V_{rem_{s,t}}$ is the value of the remembered option, *d*_*s*_ is the scale parameter and *γ*_*s*_ the memory bias parameter.

Note that the starting point bias *f*(*z*) is scaled between zero and one. A value of .5 yields that there is no starting point bias. A value > .5 indicates that there was a bias in favor of the remembered option, a value < .5 indicates that the starting point bias was favoring the forgotten option.

After fitting the model, the posterior distributions revealed that the starting point bias parameter *f*(*μ*_*z*_) did not bias decisions in favor of the remembered option (see Table 5). From the posterior estimates it is evident that, if anything, there was a small starting point bias in favor of the forgotten option.

**Table 5:** Group parameter estimates of the memory-bias-as-starting-point-bias model

Parameter	*M*	95%-HDI
*μ*_*a*_	1.77	[1.68, 1.88]
*σ*_*a*_	1.30	[1.25, 1.35]
$\mu _{T_{er}}$	0.29	[0.27, 0.31]
$\sigma _{T_{er}}$	1.46	[1.38, 1.57]
*μ*_*z*_	0.49	[0.48, 0.50]
*σ*_*z*_	1.05	[1.00, 1.08]
$\mu _{d_{SPDM}}$	0.36	[0.31, 0.41]
$\sigma _{d_{SPDM}}$	1.70	[1.51, 1.93]
*μ*_*γ*_	− 0.62	[− 0.78, − 0.45]
*σ*_*γ*_	1.82	[1.58, 2.10]

Note. *M* represents the posterior mean. The 95%-HDI depicts the boundaries of the 95% highest-density interval. Note that these values are transformed with respective transformation functions (see main text)

The model yielded a WAIC of 17,425 which was higher than the WAIC of the SPDM.
